# [μ-1,3-Bis(3,5-dimethyl-1*H*-pyrazol-1-yl-κ*N* ^2^)propan-2-olato-κ^2^ *O*:*O*]bis­[(ethanol-κ*O*)zinc(II)] bis­(perchlorate)

**DOI:** 10.1107/S160053681004479X

**Published:** 2010-11-06

**Authors:** Da-Min Tian, Chong-Yu Shi

**Affiliations:** a Department of Chemistry and Chemical Engineering, Henan University of Urban Construction, Pingdingshan, Henan 467044, People’s Republic of China; b Zhongzhou University, Zhongzhou 450044, People’s Republic of China

## Abstract

In the centrosymmetric dinuclear title complex, [Zn_2_(C_13_H_19_N_4_O)_2_(C_2_H_5_OH)_2_](ClO_4_)_2_, the Zn^II^ atom is in a distorted trigonal-bipyramidal coordination geometry. The equatorial plane is constructed by one N atom and one O atom from two 1,3-bis­(3,5-dimethyl­pyrazol-1-yl)propan-2-olate (bppo) ligands and one O atom from an ethanol mol­ecule. One N atom and one O atom from the two bppo ligands occupy the axial positions. Inter­molecular O—H⋯O hydrogen bonds between the ethanol mol­ecules and perchlorate anions, and O⋯π inter­actions between the perchlorate anions and pyrazole rings [O⋯centroid distances = 3.494 (3) and 3.413 (3) Å], lead to a chain structure along [010].

## Related literature

For related structures, see: Montoya *et al.* (2007 ▶).
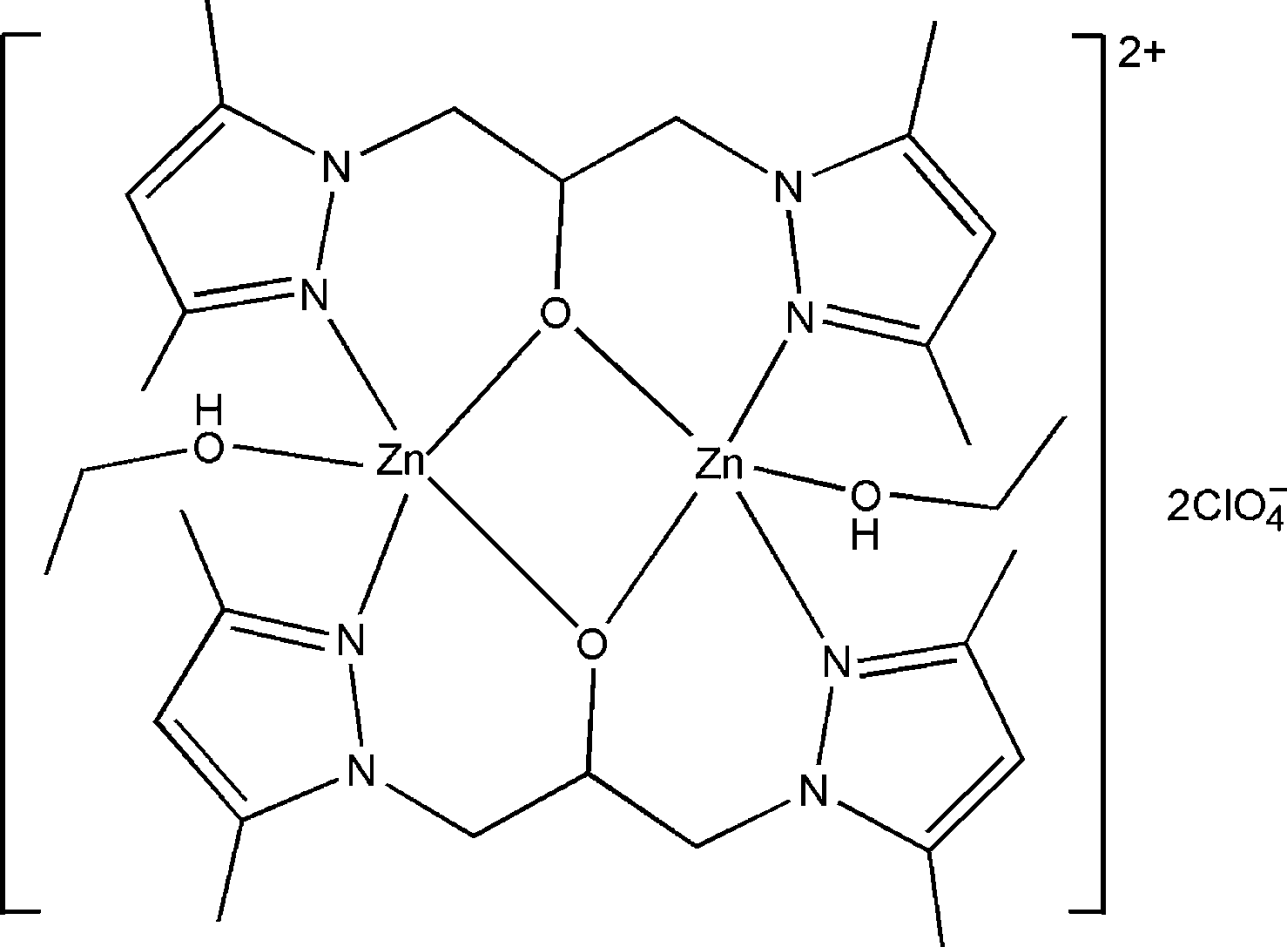


## Experimental

### 

#### Crystal data

[Zn_2_(C_13_H_19_N_4_O)_2_(C_2_H_6_O)_2_](ClO_4_)_2_
*M* *_r_* = 916.42
Triclinic, 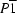

*a* = 8.8570 (18) Å
*b* = 11.148 (2) Å
*c* = 11.300 (2) Å
α = 111.13 (3)°
β = 100.40 (3)°
γ = 100.11 (3)°
*V* = 987.8 (5) Å^3^
*Z* = 1
Mo *K*α radiation
μ = 1.42 mm^−1^
*T* = 293 K
0.22 × 0.20 × 0.20 mm

#### Data collection

Bruker APEX CCD diffractometer
Absorption correction: multi-scan (*SADABS*; Sheldrick, 1996 ▶) *T*_min_ = 0.765, *T*_max_ = 0.765
7788 measured reflections
3482 independent reflections
3172 reflections with *I* > 2σ(*I*)
*R*_int_ = 0.017

#### Refinement

*R*[*F* ^2^ > 2σ(*F* ^2^)] = 0.030
*wR*(*F* ^2^) = 0.083
*S* = 1.07
3482 reflections
249 parameters
2 restraints
H-atom parameters constrained
Δρ_max_ = 0.50 e Å^−3^
Δρ_min_ = −0.22 e Å^−3^

### 

Data collection: *SMART* (Bruker, 2007 ▶); cell refinement: *SAINT* (Bruker, 2007 ▶); data reduction: *SAINT*; program(s) used to solve structure: *SHELXS97* (Sheldrick, 2008 ▶); program(s) used to refine structure: *SHELXL97* (Sheldrick, 2008 ▶); molecular graphics: *SHELXTL* (Sheldrick, 2008 ▶) and *DIAMOND* (Brandenburg, 1999 ▶); software used to prepare material for publication: *SHELXTL*.

## Supplementary Material

Crystal structure: contains datablocks I, global. DOI: 10.1107/S160053681004479X/hy2370sup1.cif

Structure factors: contains datablocks I. DOI: 10.1107/S160053681004479X/hy2370Isup2.hkl

Additional supplementary materials:  crystallographic information; 3D view; checkCIF report

## Figures and Tables

**Table 1:** Selected bond lengths (Å)

Zn1—N1	2.076 (2)
Zn1—N3^i^	2.042 (2)
Zn1—O6	1.9908 (16)
Zn1—O6^i^	2.0428 (16)
Zn1—O7	2.1292 (18)

Symmetry code: (i) 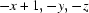.

**Table 2:** Hydrogen-bond geometry (Å, °)

*D*—H⋯*A*	*D*—H	H⋯*A*	*D*⋯*A*	*D*—H⋯*A*
O7—H7*A*⋯O3	0.85	2.03	2.860 (3)	165

**Fig. 1.**
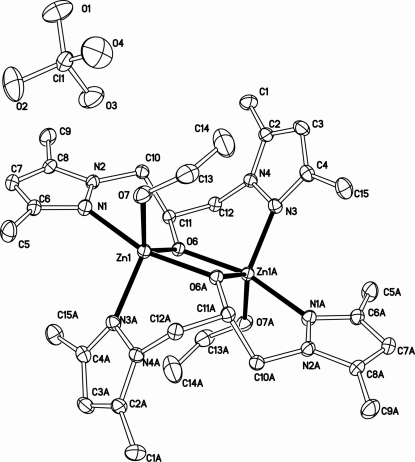
Molecular structure of the title compound. Displacement ellipsoids are drawn at the 50% probability level. H atoms have been omitted for clarity. [Symmetry code: (A) 1 - x, -y, -z.]

**Fig. 2.**
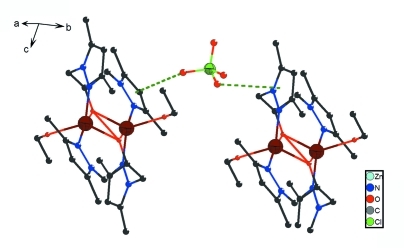
The chain structure in the title compound. Dashed lines denote O···π interactions.

## supplementary crystallographic information

### Comment

Pyrazole-derived ligands have been extensively studied in recent years. These ligands are known as anionic or neutral groups to coordinate to metal centers through N atoms in monodentate and exobidentate modes. It is essential to study the syntheses and crystal structures of the complexes formed by pyrazole systematically, and to inquire into the factors that influence the formation and structure of such complexes. Such studies may lead to the design and synthesis of functional materials, and also provide a theoretical foundation for supramolecular chemistry and crystal engineering (Montoya *et al.*, 2007). As part of our studies on the synthesis and characterization of these compounds, we report here the synthesis and crystal structure of the title compound.

In the title compound (Fig. 1), the Zn^II^ atom is five-coordinated by two O atoms and two N atoms from two 1,3-bis(3,5-dimethyl-pyrazol-1-yl)propan-2-olate (bppo) ligands and one O atom from an ethanol molecule in a distorted trigonal–bipyramidal geometry (Table 1). The equatorial plane is constructed by N3^i^ and O6 from the two bppo ligands and O7 from the ethanol molecule. The N1 and O6^i^ atoms occupy the axial positions [symmetry code: (i) 1 - x, -y, -z]. Two hydroxyl O atoms bridge the Zn atoms, forming a dinuclear complex. Intermolecular O—H···O hydrogen bonds between the ethanol molecules and perchlorate anions (Table 2) and O···π interactions between the perchlorate anions and pyrazole rings, O2···*Cg*1^ii^ and O3···*Cg*2, [*Cg*1 and *Cg*2 are the centroids of C2/C3/C4/N3/N4 ring and C6/C7/C8/N1/N2 ring; symmetry code: (ii) x, -1 + y, z; O—centroid distances = 3.494 (3) and 3.413 (3) Å, respectively], lead to a chain structure along [010] (Fig. 2).

### Experimental

1,3-Bis(3,5-dimethyl-pyrazol-1-yl)propan-2-ol and ZnCl_2_.6H_2_O were available commercially and were used without further purification. 1,3-Bis(3,5-dimethyl-pyrazol-1-yl)propan-2-ol (124 mg, 0.5 mmol) were dissolved in anhydrous alcohol (15 ml). To this solution was added ZuCl_2_.6H_2_O (122 mg, 0.5 mmol) in anhydrous alcohol (10 ml). After keeping the resulting solution in air to evaporate about half of the solvent, blue prismatic crystals of the title compound were formed. The crystals were isolated, washed with alcohol three times and dried in a vacuum desiccator using silica gel (yield: 75%). Analysis, calculated for C_30_H_50_Cl_2_N_8_O_12_Zn_2_: C 39.32, H 5.50, N, 12.23%; found: C 39.42, H 5.28, N 12.35%.

### Refinement

H atoms on C atoms were positioned geometrically and refined as riding atoms, with C—H = 0.93–0.98 Å and *U*_iso_(H) = 1.2(1.5 for methyl)*U*_eq_(C). Hydroxy H atom was located in a difference Fourier map and refined as a riding atom, with O—H = 0.85 Å and *U*_iso_(H) = 1.5*U*_eq_(O).

### Crystal data

**Table:**

[Zn_2_(C_13_H_19_N_4_O)_2_(C_2_H_6_O)_2_](ClO_4_)_2_	*Z* = 1
*M**_r_* = 916.42	*F*(000) = 476
Triclinic, *P*1	*D*_x_ = 1.541 Mg m^−^^3^
Hall symbol: -P 1	Mo *K*α radiation, λ = 0.71073 Å
*a* = 8.8570 (18) Å	Cell parameters from 2230 reflections
*b* = 11.148 (2) Å	θ = 2.3–25.7°
*c* = 11.300 (2) Å	µ = 1.42 mm^−^^1^
α = 111.13 (3)°	*T* = 293 K
β = 100.40 (3)°	Block, colourless
γ = 100.11 (3)°	0.22 × 0.20 × 0.20 mm
*V* = 987.8 (5) Å^3^	

### Data collection

**Table:**

Bruker APEX CCD diffractometer	3482 independent reflections
Radiation source: fine-focus sealed tube	3172 reflections with *I* > 2σ(*I*)
graphite	*R*_int_ = 0.017
φ and ω scans	θ_max_ = 25.0°, θ_min_ = 3.3°
Absorption correction: multi-scan (*SADABS*; Sheldrick, 1996)	*h* = −10→10
*T*_min_ = 0.765, *T*_max_ = 0.765	*k* = −13→13
7788 measured reflections	*l* = −13→13

### Refinement

**Table:**

Refinement on *F*^2^	Primary atom site location: structure-invariant direct methods
Least-squares matrix: full	Secondary atom site location: difference Fourier map
*R*[*F*^2^ > 2σ(*F*^2^)] = 0.030	Hydrogen site location: inferred from neighbouring sites
*wR*(*F*^2^) = 0.083	H-atom parameters constrained
*S* = 1.07	*w* = 1/[σ^2^(*F*_o_^2^) + (0.0486*P*)^2^ + 0.4173*P*] where *P* = (*F*_o_^2^ + 2*F*_c_^2^)/3
3482 reflections	(Δ/σ)_max_ < 0.001
249 parameters	Δρ_max_ = 0.50 e Å^−^^3^
2 restraints	Δρ_min_ = −0.22 e Å^−^^3^

### Fractional atomic coordinates and isotropic or equivalent isotropic displacement parameters (Å^2^)

**Table:**

	*x*	*y*	*z*	*U*_iso_*/*U*_eq_	
Zn1	0.44360 (3)	−0.13668 (2)	0.00434 (2)	0.03250 (11)	
N1	0.3373 (2)	−0.18995 (19)	0.13387 (19)	0.0385 (4)	
N2	0.3198 (2)	−0.09350 (19)	0.24246 (19)	0.0364 (4)	
O6	0.40890 (17)	0.04471 (14)	0.07488 (15)	0.0331 (3)	
O7	0.6260 (2)	−0.21497 (19)	0.07459 (19)	0.0528 (5)	
H7A	0.6208	−0.2359	0.1393	0.079*	
C5	0.2447 (4)	−0.4353 (3)	0.0179 (3)	0.0597 (7)	
H5A	0.2756	−0.4202	−0.0538	0.089*	
H5B	0.1397	−0.4941	−0.0135	0.089*	
H5C	0.3182	−0.4748	0.0544	0.089*	
C6	0.2460 (3)	−0.3054 (2)	0.1218 (2)	0.0413 (5)	
C7	0.1694 (3)	−0.2813 (3)	0.2213 (3)	0.0453 (6)	
H7	0.0970	−0.3442	0.2334	0.054*	
C8	0.2202 (3)	−0.1478 (3)	0.2983 (2)	0.0413 (5)	
C9	0.1855 (4)	−0.0683 (3)	0.4239 (3)	0.0606 (8)	
H9A	0.2773	−0.0431	0.4961	0.091*	
H9B	0.0971	−0.1210	0.4368	0.091*	
H9C	0.1599	0.0104	0.4193	0.091*	
C10	0.4166 (3)	0.0433 (2)	0.2899 (2)	0.0381 (5)	
H10A	0.5279	0.0435	0.3030	0.046*	
H10B	0.4016	0.0957	0.3743	0.046*	
C11	0.3753 (3)	0.1084 (2)	0.1951 (2)	0.0331 (5)	
H11	0.2605	0.0997	0.1770	0.040*	
C13	0.7924 (3)	−0.1751 (4)	0.0899 (3)	0.0641 (8)	
H13A	0.8108	−0.1760	0.0076	0.077*	
H13B	0.8423	−0.2383	0.1110	0.077*	
C14	0.8653 (5)	−0.0412 (4)	0.1945 (6)	0.1128 (18)	
H14A	0.8059	0.0190	0.1808	0.169*	
H14B	0.9729	−0.0113	0.1926	0.169*	
H14C	0.8647	−0.0439	0.2783	0.169*	
C12	0.4601 (3)	0.2576 (2)	0.2567 (2)	0.0367 (5)	
H12A	0.4222	0.2976	0.1977	0.044*	
H12B	0.4328	0.2998	0.3385	0.044*	
N3	0.7009 (2)	0.25422 (18)	0.17989 (19)	0.0366 (4)	
N4	0.6328 (2)	0.28347 (18)	0.28297 (18)	0.0359 (4)	
C1	0.7043 (4)	0.3867 (3)	0.5278 (3)	0.0630 (8)	
H1A	0.6439	0.4506	0.5295	0.094*	
H1B	0.8007	0.4283	0.5977	0.094*	
H1C	0.6428	0.3136	0.5389	0.094*	
C2	0.7442 (3)	0.3366 (2)	0.3990 (2)	0.0437 (6)	
C3	0.8885 (3)	0.3381 (3)	0.3704 (3)	0.0501 (6)	
H3	0.9877	0.3674	0.4306	0.060*	
C15	0.9736 (3)	0.2689 (3)	0.1524 (3)	0.0614 (8)	
H15A	0.9241	0.1944	0.0696	0.092*	
H15B	1.0653	0.2525	0.1974	0.092*	
H15C	1.0055	0.3479	0.1373	0.092*	
C4	0.8579 (3)	0.2875 (2)	0.2346 (3)	0.0433 (6)	
O1	0.7169 (4)	−0.2927 (3)	0.4897 (3)	0.1003 (9)	
O2	0.5741 (4)	−0.4532 (3)	0.2842 (4)	0.1233 (12)	
O3	0.6271 (4)	−0.2330 (3)	0.3209 (3)	0.0955 (9)	
O4	0.8318 (4)	−0.3383 (4)	0.3177 (3)	0.1242 (12)	
Cl1	0.68858 (8)	−0.33052 (6)	0.35237 (7)	0.05214 (18)	

### Atomic displacement parameters (Å^2^)

**Table:**

	*U*^11^	*U*^22^	*U*^33^	*U*^12^	*U*^13^	*U*^23^
Zn1	0.03703 (16)	0.02905 (16)	0.03013 (16)	0.00679 (10)	0.01162 (11)	0.01013 (11)
N1	0.0468 (11)	0.0325 (10)	0.0367 (11)	0.0091 (8)	0.0174 (9)	0.0121 (8)
N2	0.0422 (10)	0.0341 (10)	0.0325 (10)	0.0070 (8)	0.0130 (8)	0.0131 (8)
O6	0.0396 (8)	0.0292 (8)	0.0296 (8)	0.0076 (6)	0.0132 (6)	0.0099 (6)
O7	0.0477 (10)	0.0682 (12)	0.0590 (12)	0.0215 (9)	0.0158 (9)	0.0403 (10)
C5	0.078 (2)	0.0344 (14)	0.0638 (19)	0.0087 (13)	0.0274 (16)	0.0154 (13)
C6	0.0462 (13)	0.0354 (13)	0.0418 (14)	0.0060 (10)	0.0114 (11)	0.0175 (11)
C7	0.0459 (13)	0.0442 (14)	0.0467 (15)	0.0021 (11)	0.0137 (11)	0.0231 (12)
C8	0.0449 (13)	0.0474 (14)	0.0365 (13)	0.0088 (10)	0.0155 (10)	0.0218 (11)
C9	0.0747 (19)	0.0637 (18)	0.0458 (16)	0.0113 (15)	0.0313 (15)	0.0207 (14)
C10	0.0417 (12)	0.0356 (12)	0.0318 (12)	0.0043 (9)	0.0096 (10)	0.0104 (10)
C11	0.0325 (11)	0.0335 (11)	0.0330 (12)	0.0086 (9)	0.0139 (9)	0.0105 (9)
C13	0.0497 (16)	0.099 (3)	0.066 (2)	0.0353 (16)	0.0243 (15)	0.0470 (19)
C14	0.068 (2)	0.086 (3)	0.177 (5)	0.002 (2)	−0.007 (3)	0.072 (3)
C12	0.0416 (12)	0.0318 (12)	0.0366 (13)	0.0108 (9)	0.0159 (10)	0.0107 (10)
N3	0.0364 (10)	0.0339 (10)	0.0340 (10)	0.0066 (8)	0.0112 (8)	0.0079 (8)
N4	0.0401 (10)	0.0319 (10)	0.0304 (10)	0.0054 (8)	0.0095 (8)	0.0082 (8)
C1	0.080 (2)	0.0609 (18)	0.0336 (15)	0.0046 (15)	0.0084 (14)	0.0131 (13)
C2	0.0550 (15)	0.0314 (12)	0.0344 (13)	0.0021 (10)	0.0037 (11)	0.0098 (10)
C3	0.0450 (14)	0.0431 (14)	0.0467 (16)	0.0014 (11)	−0.0047 (12)	0.0134 (12)
C15	0.0406 (14)	0.0656 (19)	0.075 (2)	0.0121 (13)	0.0207 (14)	0.0229 (16)
C4	0.0392 (12)	0.0352 (13)	0.0489 (15)	0.0062 (10)	0.0078 (11)	0.0133 (11)
O1	0.135 (2)	0.135 (3)	0.0666 (17)	0.065 (2)	0.0451 (17)	0.0586 (18)
O2	0.125 (2)	0.0526 (16)	0.163 (3)	−0.0057 (15)	0.031 (2)	0.0271 (18)
O3	0.140 (2)	0.0671 (16)	0.0824 (18)	0.0311 (16)	0.0106 (17)	0.0403 (14)
O4	0.088 (2)	0.178 (3)	0.116 (3)	0.040 (2)	0.0609 (19)	0.051 (2)
Cl1	0.0664 (4)	0.0457 (4)	0.0509 (4)	0.0129 (3)	0.0249 (3)	0.0234 (3)

### Geometric parameters (Å, °)

**Table:**

Zn1—N1	2.076 (2)	C11—H11	0.9800
Zn1—N3^i^	2.042 (2)	C13—C14	1.468 (6)
Zn1—O6	1.9908 (16)	C13—H13A	0.9700
Zn1—O6^i^	2.0428 (16)	C13—H13B	0.9700
Zn1—O7	2.1292 (18)	C14—H14A	0.9600
Zn1—Zn1^i^	3.0784 (9)	C14—H14B	0.9600
N1—C6	1.340 (3)	C14—H14C	0.9600
N1—N2	1.364 (3)	C12—N4	1.460 (3)
N2—C8	1.353 (3)	C12—H12A	0.9700
N2—C10	1.461 (3)	C12—H12B	0.9700
O6—C11	1.401 (3)	N3—C4	1.341 (3)
O6—Zn1^i^	2.0428 (16)	N3—N4	1.370 (3)
O7—C13	1.422 (3)	N3—Zn1^i^	2.042 (2)
O7—H7A	0.8500	N4—C2	1.346 (3)
C5—C6	1.496 (4)	C1—C2	1.491 (4)
C5—H5A	0.9600	C1—H1A	0.9600
C5—H5B	0.9600	C1—H1B	0.9600
C5—H5C	0.9600	C1—H1C	0.9600
C6—C7	1.385 (4)	C2—C3	1.372 (4)
C7—C8	1.366 (4)	C3—C4	1.385 (4)
C7—H7	0.9300	C3—H3	0.9300
C8—C9	1.497 (4)	C15—C4	1.495 (4)
C9—H9A	0.9600	C15—H15A	0.9600
C9—H9B	0.9600	C15—H15B	0.9600
C9—H9C	0.9600	C15—H15C	0.9600
C10—C11	1.522 (3)	O1—Cl1	1.414 (3)
C10—H10A	0.9700	O2—Cl1	1.401 (3)
C10—H10B	0.9700	O3—Cl1	1.422 (3)
C11—C12	1.534 (3)	O4—Cl1	1.402 (3)
			
O6—Zn1—N3^i^	112.71 (8)	C10—C11—C12	111.32 (19)
O6—Zn1—O6^i^	80.52 (7)	O6—C11—H11	107.6
N3^i^—Zn1—O6^i^	89.87 (7)	C10—C11—H11	107.6
O6—Zn1—N1	91.69 (7)	C12—C11—H11	107.6
N3^i^—Zn1—N1	106.21 (8)	O7—C13—C14	111.5 (3)
O6^i^—Zn1—N1	163.86 (7)	O7—C13—H13A	109.3
O6—Zn1—O7	130.72 (8)	C14—C13—H13A	109.3
N3^i^—Zn1—O7	115.77 (8)	O7—C13—H13B	109.3
O6^i^—Zn1—O7	91.14 (7)	C14—C13—H13B	109.3
N1—Zn1—O7	83.23 (8)	H13A—C13—H13B	108.0
O6—Zn1—Zn1^i^	40.88 (4)	C13—C14—H14A	109.5
N3^i^—Zn1—Zn1^i^	104.37 (6)	C13—C14—H14B	109.5
O6^i^—Zn1—Zn1^i^	39.63 (4)	H14A—C14—H14B	109.5
N1—Zn1—Zn1^i^	131.04 (6)	C13—C14—H14C	109.5
O7—Zn1—Zn1^i^	115.79 (6)	H14A—C14—H14C	109.5
C6—N1—N2	106.23 (19)	H14B—C14—H14C	109.5
C6—N1—Zn1	132.19 (16)	N4—C12—C11	112.81 (18)
N2—N1—Zn1	119.85 (14)	N4—C12—H12A	109.0
C8—N2—N1	110.49 (19)	C11—C12—H12A	109.0
C8—N2—C10	129.5 (2)	N4—C12—H12B	109.0
N1—N2—C10	119.68 (18)	C11—C12—H12B	109.0
C11—O6—Zn1	126.98 (13)	H12A—C12—H12B	107.8
C11—O6—Zn1^i^	124.67 (13)	C4—N3—N4	105.54 (19)
Zn1—O6—Zn1^i^	99.48 (7)	C4—N3—Zn1^i^	134.36 (17)
C13—O7—Zn1	128.78 (17)	N4—N3—Zn1^i^	117.88 (14)
C13—O7—H7A	103.2	C2—N4—N3	111.10 (19)
Zn1—O7—H7A	118.4	C2—N4—C12	129.4 (2)
C6—C5—H5A	109.5	N3—N4—C12	119.52 (18)
C6—C5—H5B	109.5	C2—C1—H1A	109.5
H5A—C5—H5B	109.5	C2—C1—H1B	109.5
C6—C5—H5C	109.5	H1A—C1—H1B	109.5
H5A—C5—H5C	109.5	C2—C1—H1C	109.5
H5B—C5—H5C	109.5	H1A—C1—H1C	109.5
N1—C6—C7	109.4 (2)	H1B—C1—H1C	109.5
N1—C6—C5	121.1 (2)	N4—C2—C3	106.6 (2)
C7—C6—C5	129.4 (2)	N4—C2—C1	122.5 (2)
C8—C7—C6	107.2 (2)	C3—C2—C1	130.8 (2)
C8—C7—H7	126.4	C2—C3—C4	106.8 (2)
C6—C7—H7	126.4	C2—C3—H3	126.6
N2—C8—C7	106.7 (2)	C4—C3—H3	126.6
N2—C8—C9	123.0 (2)	C4—C15—H15A	109.5
C7—C8—C9	130.2 (2)	C4—C15—H15B	109.5
C8—C9—H9A	109.5	H15A—C15—H15B	109.5
C8—C9—H9B	109.5	C4—C15—H15C	109.5
H9A—C9—H9B	109.5	H15A—C15—H15C	109.5
C8—C9—H9C	109.5	H15B—C15—H15C	109.5
H9A—C9—H9C	109.5	N3—C4—C3	109.9 (2)
H9B—C9—H9C	109.5	N3—C4—C15	121.5 (2)
N2—C10—C11	112.67 (19)	C3—C4—C15	128.6 (2)
N2—C10—H10A	109.1	O2—Cl1—O4	110.6 (2)
C11—C10—H10A	109.1	O2—Cl1—O1	110.3 (2)
N2—C10—H10B	109.1	O4—Cl1—O1	109.0 (2)
C11—C10—H10B	109.1	O2—Cl1—O3	107.9 (2)
H10A—C10—H10B	107.8	O4—Cl1—O3	110.9 (2)
O6—C11—C10	111.91 (18)	O1—Cl1—O3	108.07 (18)
O6—C11—C12	110.58 (18)		
			
O6—Zn1—N1—C6	144.8 (2)	C10—N2—C8—C7	−174.6 (2)
N3^i^—Zn1—N1—C6	30.5 (2)	N1—N2—C8—C9	176.0 (2)
O6^i^—Zn1—N1—C6	−154.6 (2)	C10—N2—C8—C9	3.1 (4)
O7—Zn1—N1—C6	−84.4 (2)	C6—C7—C8—N2	2.3 (3)
Zn1^i^—Zn1—N1—C6	157.08 (19)	C6—C7—C8—C9	−175.3 (3)
O6—Zn1—N1—N2	−18.00 (17)	C8—N2—C10—C11	−120.3 (3)
N3^i^—Zn1—N1—N2	−132.25 (17)	N1—N2—C10—C11	67.4 (3)
O6^i^—Zn1—N1—N2	42.6 (3)	Zn1—O6—C11—C10	17.7 (2)
O7—Zn1—N1—N2	112.83 (18)	Zn1^i^—O6—C11—C10	−122.81 (16)
Zn1^i^—Zn1—N1—N2	−5.7 (2)	Zn1—O6—C11—C12	142.41 (15)
C6—N1—N2—C8	0.5 (3)	Zn1^i^—O6—C11—C12	1.9 (2)
Zn1—N1—N2—C8	167.30 (16)	N2—C10—C11—O6	−64.4 (2)
C6—N1—N2—C10	174.2 (2)	N2—C10—C11—C12	171.27 (17)
Zn1—N1—N2—C10	−19.0 (3)	Zn1—O7—C13—C14	67.8 (4)
N3^i^—Zn1—O6—C11	126.18 (16)	O6—C11—C12—N4	−60.2 (2)
O6^i^—Zn1—O6—C11	−147.97 (19)	C10—C11—C12—N4	64.9 (2)
N1—Zn1—O6—C11	17.82 (17)	C4—N3—N4—C2	1.5 (2)
O7—Zn1—O6—C11	−64.70 (18)	Zn1^i^—N3—N4—C2	166.94 (15)
Zn1^i^—Zn1—O6—C11	−147.97 (19)	C4—N3—N4—C12	−179.55 (19)
N3^i^—Zn1—O6—Zn1^i^	−85.85 (8)	Zn1^i^—N3—N4—C12	−14.1 (2)
O6^i^—Zn1—O6—Zn1^i^	0.0	C11—C12—N4—C2	−111.0 (3)
N1—Zn1—O6—Zn1^i^	165.79 (8)	C11—C12—N4—N3	70.2 (3)
O7—Zn1—O6—Zn1^i^	83.27 (10)	N3—N4—C2—C3	−1.9 (3)
O6—Zn1—O7—C13	−62.0 (3)	C12—N4—C2—C3	179.3 (2)
N3^i^—Zn1—O7—C13	106.8 (2)	N3—N4—C2—C1	175.6 (2)
O6^i^—Zn1—O7—C13	16.4 (2)	C12—N4—C2—C1	−3.3 (4)
N1—Zn1—O7—C13	−148.4 (2)	N4—C2—C3—C4	1.5 (3)
Zn1^i^—Zn1—O7—C13	−15.8 (2)	C1—C2—C3—C4	−175.6 (3)
N2—N1—C6—C7	1.0 (3)	N4—N3—C4—C3	−0.5 (3)
Zn1—N1—C6—C7	−163.53 (18)	Zn1^i^—N3—C4—C3	−162.39 (18)
N2—N1—C6—C5	−175.6 (2)	N4—N3—C4—C15	179.5 (2)
Zn1—N1—C6—C5	20.0 (4)	Zn1^i^—N3—C4—C15	17.6 (4)
N1—C6—C7—C8	−2.0 (3)	C2—C3—C4—N3	−0.7 (3)
C5—C6—C7—C8	174.1 (3)	C2—C3—C4—C15	179.4 (3)
N1—N2—C8—C7	−1.8 (3)		

Symmetry codes: (i) −*x*+1, −*y*, −*z*.

### Hydrogen-bond geometry (Å, °)

**Table:**

*D*—H···*A*	*D*—H	H···*A*	*D*···*A*	*D*—H···*A*
O7—H7A···O3	0.85	2.03	2.860 (3)	165
